# Conservative Management of a Forearm Refracture With the Titanium Elastic Nailing System (TENS) In Situ in a 14-Year-Old Male: A Case Report

**DOI:** 10.7759/cureus.70646

**Published:** 2024-10-01

**Authors:** Abobaker Younis, Mehad Elmubarak

**Affiliations:** 1 Orthopaedics and Traumatology, University Hospital Galway, Galway, IRL

**Keywords:** clinical case report, galway, ireland, pediatric forearm fracture, tens tensile elastic nail system

## Abstract

This case report presents a 14-year-old male patient with a medical history of left nephrectomy for cystic nephroma and resolved hypertension, who sustained a right both-bone middle shaft forearm fracture while playing football. The injury was managed initially with manipulation under anesthesia, the insertion of the Titanium Elastic Nailing System (TENS), and the application of an above-elbow cast. Clinical and radiographic reviews confirmed fracture healing after seven weeks, allowing for cast removal and advising the patient to avoid contact sports. However, 10 weeks post-initial treatment, the patient experienced a refracture following another traumatic incident. The refracture was treated conservatively with a combination of above-elbow and below-elbow casts over six weeks. Regular follow-ups every two weeks indicated satisfactory progress, leading to the removal of the TENS approximately four and a half months post-refracture. Subsequent clinical and radiographic evaluations showed complete fracture healing, with the patient regaining a full range of motion and intact neurovascular status at the final follow-up. This case highlights the potential for successful conservative management of pediatric forearm refractures with intramedullary nails in situ.

## Introduction

Forearm fractures are among the most common pediatric injuries, accounting for a significant percentage of childhood fractures. These injuries typically result from falls or sports-related activities, with both-bone forearm (BBFA) fractures being particularly prevalent. BBFA fractures involve breaks in both the radius and ulna, the two long bones in the forearm. Diaphyseal fractures, which occur in the shaft (midsection) of these bones, represent 3-15% of all pediatric fractures [1-3].

Standard management of pediatric forearm fractures often involves closed reduction and casting, but up to 25% of cases may experience displacement during follow-up, necessitating further intervention [3]. Recent trends suggest that an increasing number of forearm fractures in children are due to greater participation in sports and recreational activities [1]. In cases where closed reduction and casting are insufficient to maintain alignment, internal fixation with elastic intramedullary nailing, such as the Titanium Elastic Nailing System (TENS), is often favored due to its minimally invasive approach and ability to maintain bone alignment while allowing some flexibility during the healing process [4,5].

However, despite the overall effectiveness of TENS, refractures in the presence of intramedullary nails remain a rare but challenging complication. Studies have shown that mid-shaft fractures are more prone to refracture compared to distal forearm fractures, with refracture rates reaching up to 15% for diaphyseal BBFA fractures [6,7]. Several factors contribute to refracture, including incomplete fracture remodeling, early cast removal, and residual bone angulation [7-9]. Children aged 10 and older are particularly susceptible to these secondary injuries following initial treatment [9].

Although surgical intervention is often required to manage forearm refractures, conservative treatment remains a viable option in select cases, especially when displacement is minimal and alignment is preserved. This report discusses the case of a 14-year-old patient who sustained a refracture of the forearm with TENS in situ and was successfully treated using conservative measures. This case highlights the potential for non-operative management in pediatric refractures, contributing to the growing body of literature advocating for tailored treatment strategies based on individual clinical presentations.

## Case presentation

**Figure 1:**
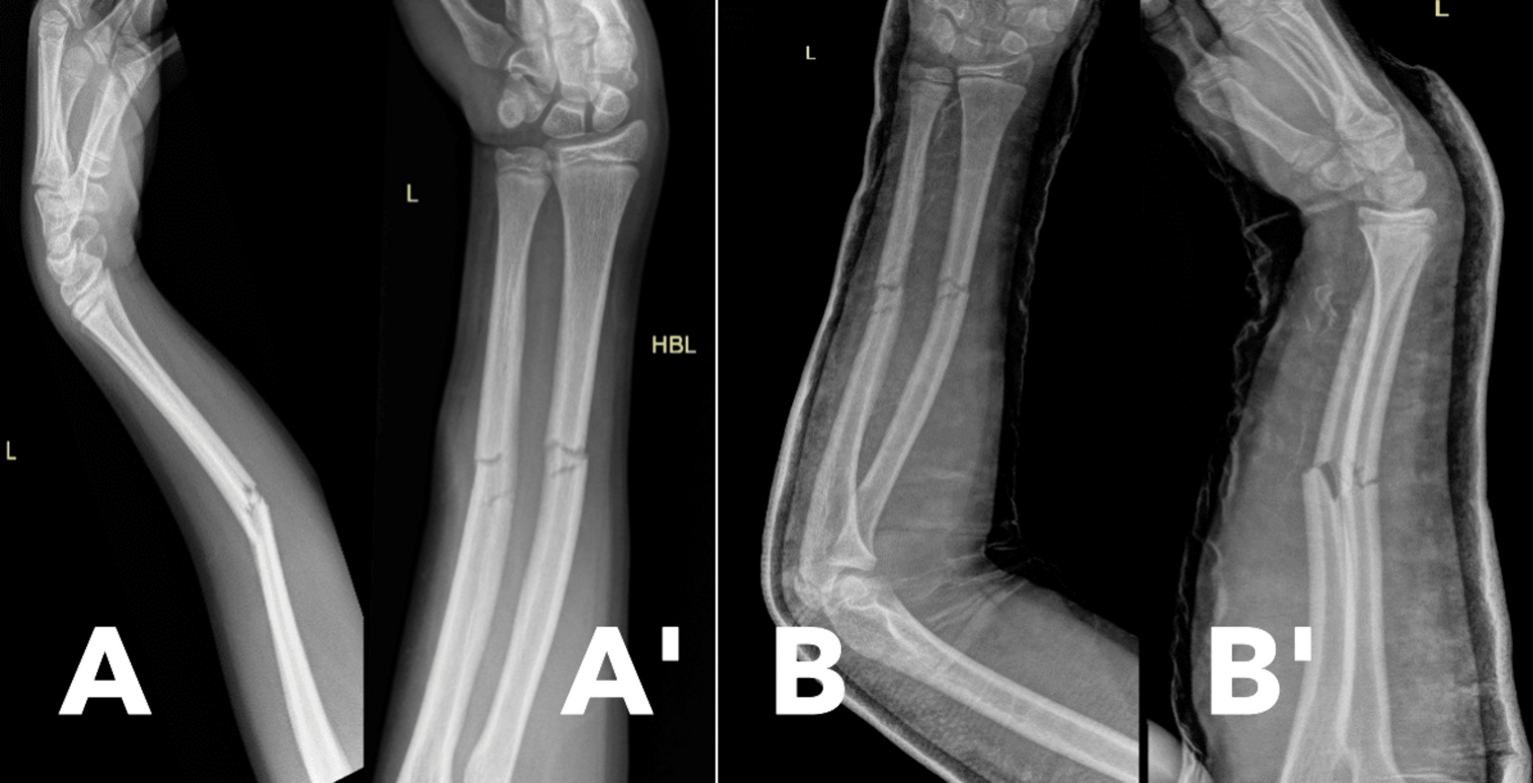
Initial radiographs (A) Lateral view of initial fracture; (A') AP view of initial fracture; (B) AP view of initial fracture after application of a back slab; (B') Lateral view of initial fracture after application of a back slab

A 14-year-old boy with a past medical history of left nephrectomy for cystic nephroma and resolved hypertension presented to the Emergency Department with a painful and deformed left forearm. The injury occurred while he was playing football; he recalled that an opponent grabbed his hand from behind, resulting in immediate, severe pain and deformity. He described the pain as sharp and intense, which worsened with any movement of the forearm. Upon examination in the emergency department, there was visible swelling and an S-shaped deformity of the forearm. However, no open wounds were noted, and the skin appeared normal in color and temperature. Neurovascular examination revealed intact sensation and motor function in the median, ulnar, and radial nerves, with palpable distal pulses. There were no other associated injuries. A back slab was applied for temporary stabilization, and he was seen by the orthopedic team for further management (Figure 1).

**Figure 2:**
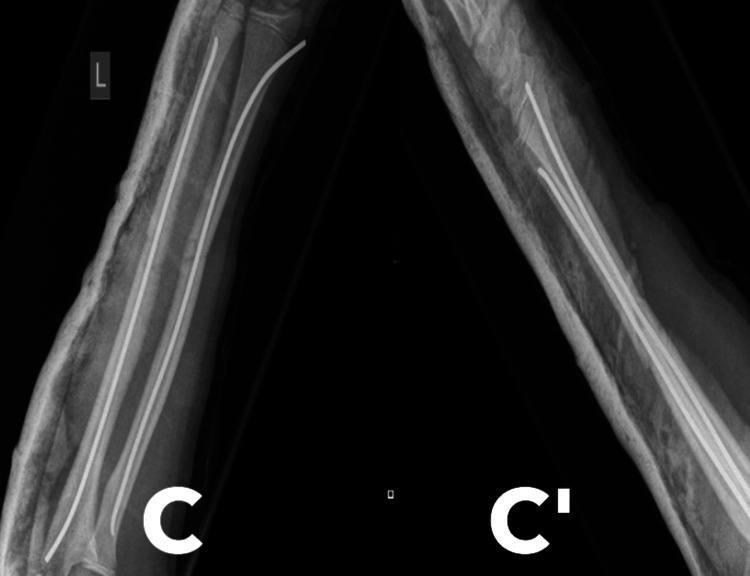
Initial operative management with TENS (C) AP view post-initial TENS application; (C') Lateral view post-initial TENS application TENS: Titanium elastic nailing system

Initial treatment consisted of manipulation under anesthesia, insertion of TENS, and application of an above-elbow cast (Figure 2). Clinical and radiographic reviews were conducted at two, four, and seven weeks. At the four-week follow-up, the above-elbow cast was replaced with a below-elbow cast. Radiological and clinical fracture healing was confirmed after seven weeks, and the cast was removed. The patient was advised to avoid contact sports and was scheduled for a follow-up visit in four months to discuss the potential removal of the metalwork.

**Figure 3:**
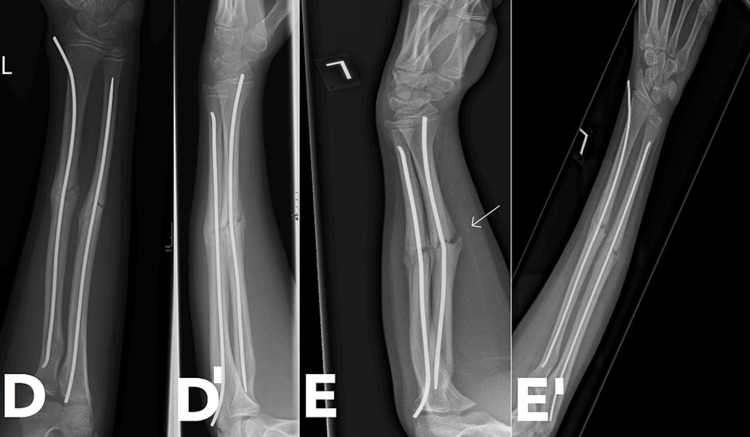
Follow up radiographs showing the refracture (D) AP view before refracture, note callus bridging the fracture; (D') Lateral view before refracture, note callus bridging the fracture; (E) Lateral view showing refracture, right arrow points to break in callus and bending of TENS in situ; (E') AP view showing refracture, no significant displacement in this view TENS: Titanium elastic nailing system

At the 10-week mark post-operatively, the patient sustained a refracture while playing when another child pulled on his left hand. He presented to the outpatient clinic a week later with tenderness around the fracture site. The patient reported being unable to perform activities requiring wrist movement, such as lifting or rotating the forearm. Plain radiographs confirmed a fracture of the newly formed callus (Figure 3). There was no significant displacement, but a break in the callus was evident, along with some bending of the TENS in situ. The treatment approach involved applying an above-elbow cast for four weeks, followed by a below-elbow cast for an additional two weeks. The patient was instructed to mobilize after cast removal but was advised to avoid contact sports for at least six more weeks.

**Figure 4:**
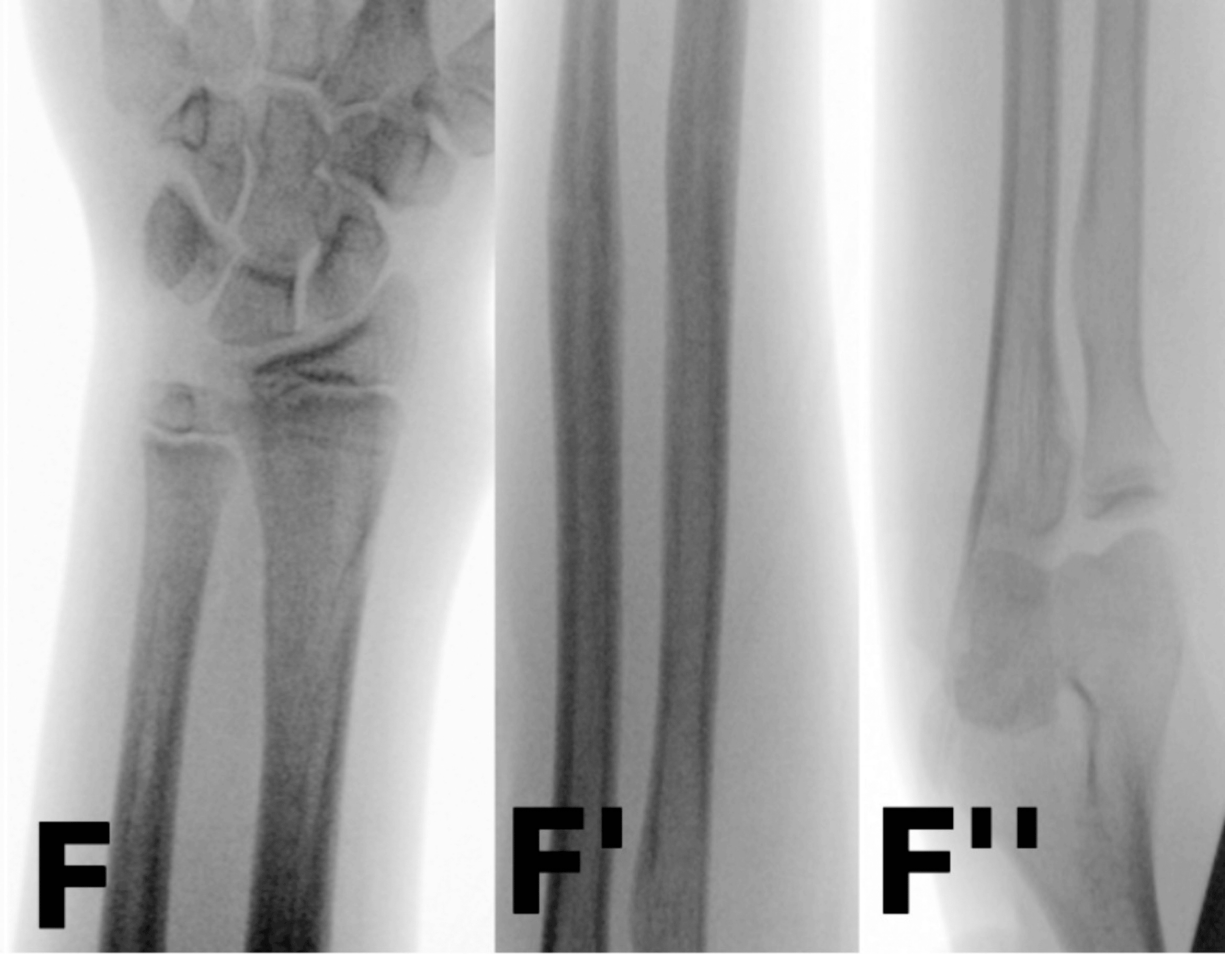
Intraoperative radiographs showing uncomplicated removal of TENS AP view of the wrist (F), forearm shaft (F'), and proximal forearm (F'') during TENS removal TENS: Titanium elastic nailing system

The patient was monitored in the clinic every two weeks. The TENS was removed approximately four and a half months post-refracture (Figure 4). He was seen again at the one- and three-week follow-ups after TENS removal. Clinical and radiographic assessments confirmed that the fracture had completely healed. At his final follow-up, the patient demonstrated a full range of motion in the left forearm and intact motor and sensory function in the median, radial, ulnar, anterior interosseous, and posterior interosseous nerves.

## Discussion

The presented case of a 14-year-old boy with a forearm refracture, managed conservatively with TENS in situ, provides valuable insights into the management of pediatric forearm fractures, particularly those involving refractures. This case aligns with the experiences reported in similar studies, underscoring both the challenges and potential benefits of conservative treatment approaches in pediatric patients.

The initial management of the patient's forearm fracture, using TENS and a cast, was in accordance with widely accepted practice for pediatric forearm fractures. Internal fixation with TENS is favored due to its minimally invasive nature and ability to maintain alignment while allowing for some degree of flexibility [4]. This approach has been documented to result in favorable outcomes in most cases, as it did initially in our patient, with clinical and radiographic healing confirmed after seven weeks [2,4].

However, the occurrence of a refracture at 10 weeks post-initial treatment presented a significant challenge. Refracture in the presence of intramedullary nails, while rare, has been documented in the literature. For instance, Mittal et al. reported on the 'failure' of forearm intramedullary elastic nails, highlighting the potential for such complications even with initially successful interventions [8]. In our case, the refracture occurred due to another traumatic incident, which aligns with findings that suggest additional trauma is a common cause of refracture [7].

Our approach to managing the refracture conservatively - by reapplying an above-elbow cast followed by a below-elbow cast - aimed to provide stability while allowing for the natural healing process. This method contrasts with some cases where surgical re-intervention is chosen. For instance, van Egmond et al. described cases where refractures with nails in situ were managed through surgical re-intervention, emphasizing the variability in treatment approaches depending on the specific clinical scenario [6].

The decision to avoid further surgical intervention in our patient was influenced by several factors, including the patient’s overall health, the nature of the refracture, and the preference to minimize additional trauma and potential complications associated with surgery. This conservative approach has been supported by some studies, which suggest that, in the absence of significant displacement or misalignment, conservative management can be effective [9,10]. Our patient’s successful outcome, with a full range of motion and no neurological deficits at the final follow-up, supports this approach.

Comparing our case to other reported cases in the literature, several case series provide valuable context. Fernandez et al. reported on a large series of pediatric patients with forearm refractures treated with both conservative and surgical approaches. Their findings indicated that, while surgical intervention was often necessary for significant refractures, conservative treatment could be successfully employed in cases without severe displacement [11]. Similarly, O'Neill et al. described a six-year-old girl with a refracture managed conservatively with good outcomes, emphasizing the importance of considering non-surgical options when feasible [12]. Kapadia et al. also provided insights into the characteristics of forearm refractures in adolescents, highlighting that mid-shaft fractures are more prone to refracture and can often be managed conservatively, as was the case with our patient [13].

## Conclusions

In conclusion, our case report contributes to the growing body of evidence supporting the conservative management of pediatric forearm refractures with intramedullary nails in situ. While surgical intervention remains a critical tool for managing complex cases, conservative methods should not be overlooked, especially when considering patient-specific factors and the potential for excellent functional outcomes. Further research and the accumulation of case reports will help refine the criteria for selecting the most appropriate treatment modalities for these challenging cases.
